# Optical Property Simulations of Gold and Silver Nanostructured Arrays Within a Liquid Crystal Environment

**DOI:** 10.3390/ma18174046

**Published:** 2025-08-29

**Authors:** Zhenzhen Shang, Guoting Zhang, Xiaoying Liu, Haishen Huang

**Affiliations:** 1 School of Science, Qiongtai Normal University, Haikou 571127, China; enenshang.851117@163.com (Z.S.);; 2 Faculty of Teacher Education, Hainan Normal University, Haikou 571158, China; 3 School of Physics and Electronic Science, Zunyi Normal University, Zunyi 563006, China

**Keywords:** liquid crystal, localized surface plasmon resonance, LSPR wavelength, LSPR sensitivity

## Abstract

Tunability of the localized surface plasmon resonance (LSPR) peak position of gold and silver nanoparticle arrays embedded in a liquid crystal cell is investigated in this paper. The extinction spectra are computed using the Finite-Difference Time Domain (FDTD) simulation algorithms. Results show that the LSPR properties exhibit significant dependence on nanoparticle size and shape, array periodicity, and liquid crystal layer thickness. Notably, the LSPR wavelength saturates when the liquid crystal thickness exceeds a critical value. Furthermore, controlled rotation of the liquid crystal optical axis within distinct planes (xoy and xoz) reveals systematic variations in LSPR characteristics. Finally, we identify the key factors governing the LSPR spectral sensitivity of these noble metal nano-arrays.

## 1. Introduction

Under excitation by pump light, noble metal nanoparticles exhibit strong absorption peaks in the visible light range, which arise from the collective excitation state formed by the interaction between free electrons on the metal surface and the positive ion background. This excited state can interact strongly with the optical field, generating a particularly strong electric field surrounding the noble metal and inducing localized surface plasmon resonance (LSPR) [1,2,3,4]. This resonance plays a crucial role in modulating other optical effects. For example, the LSPR effect can localize the strong optical field within the waveguide structures and facilitate light transmission. In random lasers, the LSPR effect can enhance the localized field around noble metal nanoparticles and interact with fluorescent molecules, ultimately enhancing the photoluminescence of the dye molecules [5]. Moreover, this effect can modify the distribution of charge carriers and the properties of impurity levels within semiconductors, as well as enhance nonlinear optical effects such as frequency doubling, mixing, and optical limiting [5,6,7]. The unique spectral characteristics of LSPR have attracted widespread research interest, leading to broad applications in fields such as biosensing, colorimetric sensing, optical sensing, photocatalysis, optical information processing, and optical communication [8,9,10,11,12,13].

Research has shown that noble metal nanoparticles exhibit larger scattering cross-sections compared to conventional dielectric particles of similar sizes. The resonance frequency and field enhancement associated with LSPR are significantly influenced by the material composition, external morphology, and surrounding environment of the noble metal nanostructures [14,15,16,17,18,19]. LSPR effects are particularly pronounced on the surfaces of noble metals, especially rough surfaces, and sharp nanostructures demonstrate stronger localized field enhancements compared to non-sharp structures [20,21,22]. Currently, extensive research exists on the LSPR characteristics of noble metal nanostructures embedded in isotropic media [23,24].

However, in the development of micro-nano devices, the study of LSPR properties of metal nanoparticles in complex and anisotropic environments is of significant importance. Liquid crystals (LCs) represent a unique state of matter between solids and liquids, characterized by distinct molecular ordering and resultant anisotropic properties [25,26,27]. Crucially, their refractive index is anisotropic, meaning it varies depending on the orientation of the liquid crystal optic axis and molecular alignment. Furthermore, LCs exhibit effects such as birefringence, polarization rotation, and dispersion. Therefore, a comprehensive exploration of the LSPR characteristics of diverse noble metal nanostructures within a liquid crystal environment holds considerable significance for advancing future LSPR-based applications. Extensive research has been conducted on the LSPR properties of precious metal nanostructures within liquid crystalline media. Guey Fuh et al. found that the thermal effect of the absorption of light caused by the LSPR of gold nanoparticles enhances the optical Kerr constant of the liquid crystals [28]. Delphine and co-workers synthesized linear self-assembly of nanoparticles within LCs [29]. Xie et al. applied an electric field to LCs to change the molecular alignment of LCs and achieved feasible tuning of the LSPR of novel gold island film immersed in the LCs [30]. Recently, Wang et al. investigated the plasmon coupling of gold nanoparticle dimers dispersed in a nematic liquid crystal matrix using the polarization z-scan technique, shedding light on the intricate interplay between plasmonic nanostructures and liquid crystal matrices [31].

To select precious metal nanostructures with more readily tunable LSPR, pre-experimental simulations are critically important. Through numerical modeling, we can identify metal particles that are both fabrication-friendly and highly tunable, thereby guiding the development of micro–nano photonic devices. The Finite-Difference Time Domain (FDTD) method is a widely used numerical technique for simulating electromagnetic wave propagation in both space and time [32,33]. In this study, we employ FDTD to investigate the LSPR of a noble metal nano-array embedded within a liquid crystal environment and sandwiched by two substrates. By varying the shape, period, and external environment of the array, the LSPR of the array are controlled. Furthermore, a comparative analysis of the LSPR properties between gold and silver nano-arrays is presented. These findings provide a theoretical foundation for the future development of LSPR-based nanophotonic devices.

## 2. Model Overview

**Figure 1:**
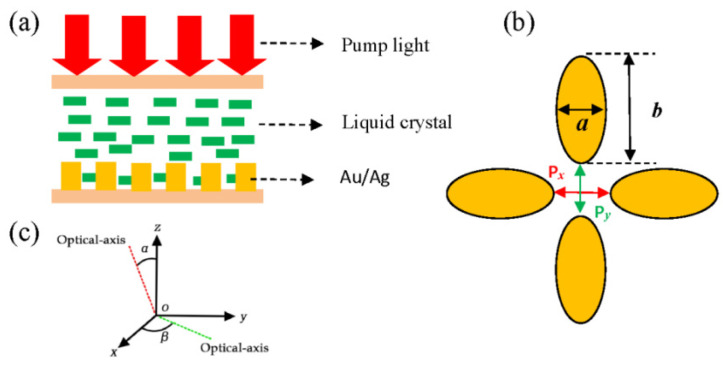
(**a**) Simulation model diagram. (**b**) Cross-sectional diagram of the noble metal nano-array. (**c**) Distribution of liquid crystal optic axis in the xoy and xoz planes.

In our simulations, noble metals (gold or silver) with elliptical aggregation structures are embedded in liquid crystal (LC) and then sandwiched between two glass substrates. Figure 1a illustrates the simulation model. Linearly polarized white pump light is vertically incident on the structure. The cross-sectional geometry of the noble metal nanostructure within the liquid crystal environment is detailed in Figure 1b, with key dimensional parameters labeled. The height of the noble metal nanostructure is 50 nm in all simulations of the paper, while the liquid crystal layer thickness is denoted by the parameter “h”. The array periodicity, identical in both x and y directions, is represented by “T”.

During the simulation computation, periodic boundary conditions were implemented in the x and y directions, while a uniaxial perfectly matched layer (UPML) absorbing boundary condition was applied in the z direction to deal with the noble metal nanoparticles. The spatial discretization was set to Δx=Δy=Δz= 3 nm, and the time step was expressed as follows:

(1) $$\Delta t={\left(c\sqrt{1/\left[{\left(\Delta x\right)}^{2}+{\left(\Delta y\right)}^{2}+{\left(\Delta z\right)}^{2}\right]}\right)}^{-1}$$

where *c* represents the speed of light. The simulated liquid crystals were of the nematic type, with ordinary and extraordinary refractive indices of $n_{o}$ = 1.53 and $n_{e}$ = 1.74, respectively.

As defined in Figure 1c, orientations of LC molecules are assumed in the xoz or xoy planes, and they may rotate around the *z*-axis or *x*-axis. The orientation of the LC molecules is characterized by two angles: α, the angle with the *z*-axis, and β, the angle with the *x*-axis. Both angles are independently adjustable during simulations. The dielectric tensor of the liquid crystal can be represented as [20]

(2) $$\varepsilon_{m}=\left[\begin{array}{ccc} {n_{o}}^{2}\;\cos^{2}\alpha +n_{e}^{2}\;\sin^{2}\alpha & 0 & (n_{o}^{2}-n_{e}^{2})\sin\alpha \cos\alpha \\ 0 & n_{o}^{2} & 0\\ (n_{o}^{2}-n_{e}^{2})\sin\alpha \cos\alpha & 0 & n_{o}^{2}\;\sin^{2}\alpha +n_{e}^{2}\;\cos^{2}\alpha \end{array}\right]$$

and

(3) $$\varepsilon_{m}=\left[\begin{array}{ccc} {n_{e}}^{2}\;\cos^{2}\beta +n_{o}^{2}\;\sin^{2}\beta & (n_{o}^{2}-n_{e}^{2})\sin\alpha \cos\alpha & 0\\ (n_{o}^{2}-n_{e}^{2})\sin\beta \cos\beta & n_{e}^{2}\;\sin^{2}\beta +n_{o}^{2}\;\cos^{2}\beta & 0\\ 0 & 0 & n_{o}^{2} \end{array}\right]$$

When the liquid crystal optical axis is in the xoz plane and xoy plane, respectively. Thus, the alteration of the orientation angle, resulting from the in-plane rotation of the liquid crystal’s optical axis, induces a modification of the dielectric tensor. The noble metal structures in the model are characterized by the Drude metal dispersion model, and their corresponding dielectric constants can be represented as follows [34]:

(4) $$\varepsilon \left(\omega \right)=\varepsilon^{\prime }+i\varepsilon^{\prime \prime }=\varepsilon_{b}+\frac{{\omega_{p}}^{2}}{-\omega^{2}-i\tau \omega }$$

where ω, τ, and $\omega_{p}$ represent the optical frequency, the damping coefficient of bound electron motion, and the bulk metal plasma frequency, respectively. $\varepsilon_{b}$ is the contribution of bound electrons in the metal. In the simulations conducted in this study, we varied the structural parameters and the orientation of LC, simulated the propagation of pump light in different structures, obtained the extinction spectra, and derived the LSPR wavelength based on the extinction spectra.

## 3. Results and Discussion

### 3.1. Extinction Spectrum Analysis

**Figure 2:**
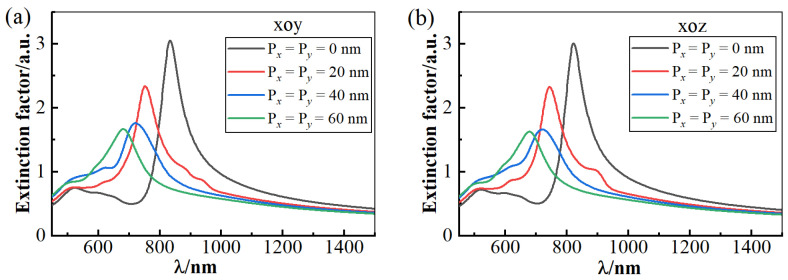
The extinction spectra of the gold nanocolumn array structure with different Px and Py when the liquid crystal optical axis is, respectively, in the (**a**) xoy plane and (**b**) xoz plane. The parameters for the structure are set as follows: a = 50 nm, b = 80 nm, h = 110 nm, T = 300 nm, α = 45°, and β = 45°.

As shown in Figure 2, the extinction spectra of the gold nanocolumn array with varying Px and Py are presented. The dimensions of the structure in the figure are a = 50 nm, b = 80 nm, liquid crystal (LC) thickness h = 110 nm, and structure period T = 300 nm. Figure 2a depicts the data when the liquid crystal optical axis lies in the xoy plane, while Figure 2b corresponds to the case where the optical axis is in the xoz plane. It is observed that, irrespective of the LC optical axis orientation (xoy or xoz plane), the extinction spectra of the gold array exhibit a significant blue shift as the parameters Px and Py increase. This spectral shift is attributed to the quantum size effect within the gold array structures. When the dimensions of the gold array structures are adjusted, the ratio of surface atoms to the total volume of the structure changes rapidly. This change induces the redistribution of free electrons between the surface and bulk of the gold array structure, consequently leading to a shift (either red or blue) in the wavelength of the localized surface plasmon resonance (LSPR).

### 3.2. LSPR Wavelength with Different Liquid Crystal Thicknesses

**Figure 3:**
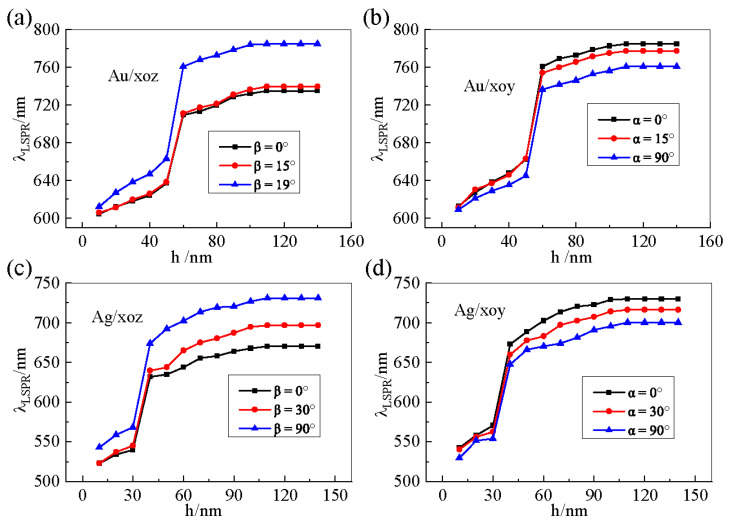
The LSPR wavelength of the gold and silver array structures in different liquid crystal environments with varying thicknesses when the liquid crystal optical axis rotates in the xoy and xoz planes for a = 50 nm, b = 80 nm, and Px = Py = 40 nm. (**a**) Gold array, optical axis in the xoy plane. (**b**) Gold array, optical axis in the xoz plane. (**c**) Silver array, optical axis in the xoy plane. (**d**) Silver array, optical axis in the xoz plane.

To study the influence of LC thickness (h) on LSPR peak position, we plot the LSPR wavelength as a function of LC thickness with the nanostructure parameters a = 50 nm, b = 80 nm, Px = 40 nm, and period T = 300 nm in Figure 3 for gold and silver nano-arrays. For the gold nano-array, we can observe from Figure 3a,b that when the LC optical axis lies in either the xoy or xoz plane, the LSPR wavelength exhibits a similar dependence on thickness, which redshifts with increasing LC thickness. Notably, once the LC thickness exceeds 100 nm, the LSPR wavelength position stabilizes and no longer shifts with further increases in thickness. This saturation behavior indicates that the distance from the top surface of the nanostructure array to the upper glass–liquid crystal interface approaches the evanescent wave damping length associated with the localized surface plasmon [20,21].

The results for the silver nano-array, presented in Figure 3c,d, reveal a following consistent trend: the LSPR wavelength also redshifts with increasing LC thickness until h ≈ 100 nm, beyond which the wavelength position remains constant. The threshold diameter h for an abrupt LSPR shift is observed at ~60 nm for Au nanoparticles (Figure 3a,b), contrasting with a significantly lower transition size of ~40 nm for Ag nanoparticles (Figure 3c,d). This occurs because in thinner liquid crystal layers, anchoring effects induce a molecular orientation near the substrate that differs from the bulk phase. This creates an inhomogeneous refractive index field around the nanoparticles, whose gradient perturbs the local electromagnetic field distribution [35]. Simultaneously, silver nanoparticles exhibit stronger local field enhancement than gold nanoparticles under near-field interactions.

### 3.3. LSPR Wavelength Variation with the Liquid Crystal Optical Axis Rotation

**Figure 4:**
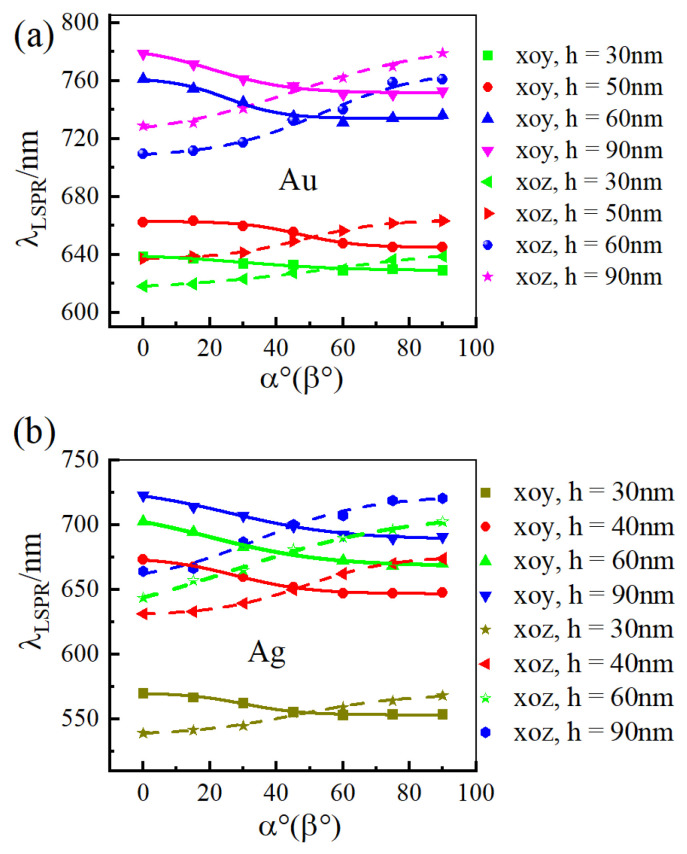
The variation of LSPR wavelength in the noble metal nano-array structures as the liquid crystal optical axis rotates within different liquid crystal layer thicknesses when the noble metal is (**a**) gold and (**b**) silver for a = 50 nm, b = 80 nm, Px = Py = 40 nm, and T = 300 nm.

Figure 4 illustrates the shift of LSPR wavelength in a noble metal nano-array as the liquid crystal (LC) optical axis rotates within the LC layer for different layer thicknesses. The nano-array material is gold in Figure 4a and silver in Figure 4b. The results show that for both gold and silver nano-arrays, rotating the LC optical axis within the xoy plane (α) from 0° to 90° induces a blue shift in the LSPR wavelength. Conversely, rotation (β) within the xoz plane over the same angular range results in a red shift. Experiments by other research groups have revealed that the rotation of the liquid crystal optical axis signifies changes in molecular alignment. This continuous variation induces a spatial averaging effect on the interaction between noble metal nanoparticles and incident light, thereby modulating the plasmon resonance response of the nanoparticles [31].

For isolated metallic nanostructures, the plasmon resonance condition is given by [16]:

(5) $$\varepsilon^{\prime }=-\kappa \varepsilon_{m}$$

where $\varepsilon_{m}$ is the dielectric constant of the surrounding environment around the metal, and κ is a shape factor of the metallic nanostructure.

Equation (5) clearly shows that the plasmon resonance conditions are related to the surrounding medium. Thereby, rotating the liquid crystal optical axis effectively changes the surrounding medium of the metallic array, leading to a redshift or blueshift of the LSPR wavelength. Specifically, when pump light illuminates the surface of the metallic nanostructure array, changes in the dielectric constant of the surrounding environment alter the amount of polarized charges. This, in turn, affects the coulombic forces governing the electron oscillations within the nanostructure, thereby changing the frequency of LSPR and causing a redshift or blueshift of the LSPR wavelength [36].

**Figure 5:**
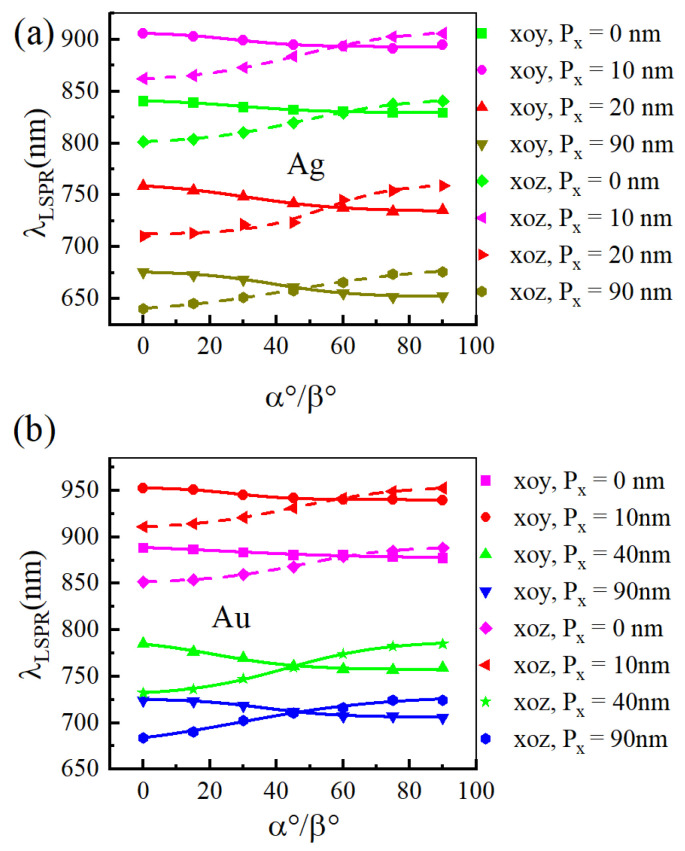
The variation of LSPR wavelength in the noble metal nano-array structures as the liquid crystal optical axis rotates within different array size when the noble metal is (**a**) gold and (**b**) silver for a = 50 nm, b = 80 nm, T = 300 nm, and h = 110 nm.

Furthermore, we computed the corresponding LSPR wavelength variations during the rotation of the liquid crystal optical axis within the xoy and xoz planes for various array size (a = 50 nm, b = 80 nm, T = 300 nm, and h = 110 nm). Figure 5 illustrates the results, which exhibit similar trends to those observed in Figure 4. For both gold and silver nano-array structures at different size, when the liquid crystal optical axis rotates within the xoy plane (α) from 0° to 90°, the LSPR wavelength exhibits a blue shift, shifting from longer to shorter wavelengths. Conversely, when the optical axis rotates within the xoz plane (β) from 0° to 90°, the LSPR wavelength undergoes a red shift, shifting from shorter to longer wavelengths.

### 3.4. LSPR Spectral Sensitivity

Thus, we can obtain the following conclusion [17]:

(6) $$\lambda_{LSPR}=\frac{2\pi c}{\omega_{p}}\sqrt{\varepsilon_{b}+\kappa \varepsilon_{m}}$$

Based on the available data from Figure 4 and Figure 5, we can discover the fitting function that describes the relationship:

(7) $$\lambda_{LSPR}=\lambda_{0}+\Delta \lambda_{LSPR}\;\sin^{2}\xi$$

For ξ=α, $\lambda_{0}={\lambda_{LSPR}}_{\alpha =0^{o}}$, $\Delta \lambda_{LSPR}={\lambda_{LSPR}}_{\alpha =90^{o}}-{\lambda_{LSPR}}_{\alpha =0^{o}}$ and for ξ=β, $\lambda_{0}={\lambda_{LSPR}}_{\beta =0^{o}}$, $\Delta \lambda_{LSPR}={\lambda_{LSPR}}_{\beta =90^{o}}-{\lambda_{LSPR}}_{\beta =0^{o}}$

We know that the LSPR spectral sensitivity is defined as [17]:

(8) $$S=\frac{d\lambda_{LSPR}}{dn_{m}}\propto \sqrt{\kappa }$$

where $n_{m}$ is the refractive index of the surrounding medium. Based on Equation (7), it can be inferred as follows:

(9) $$S\propto \Delta \lambda_{LSPR}\cos2\xi$$

Therefore, based on Equation (9), the sensitivity of the LSPR spectrum is evaluated by the wavelength shift, represented by $\Delta \lambda_{LSPR}={\lambda_{LSPR}}_{\alpha =90^{o}}-{\lambda_{LSPR}}_{\alpha =0^{o}}$ or $\Delta \lambda_{LSPR}={\lambda_{LSPR}}_{\beta =90^{o}}-{\lambda_{LSPR}}_{\beta =0^{o}}$.

**Figure 6:**
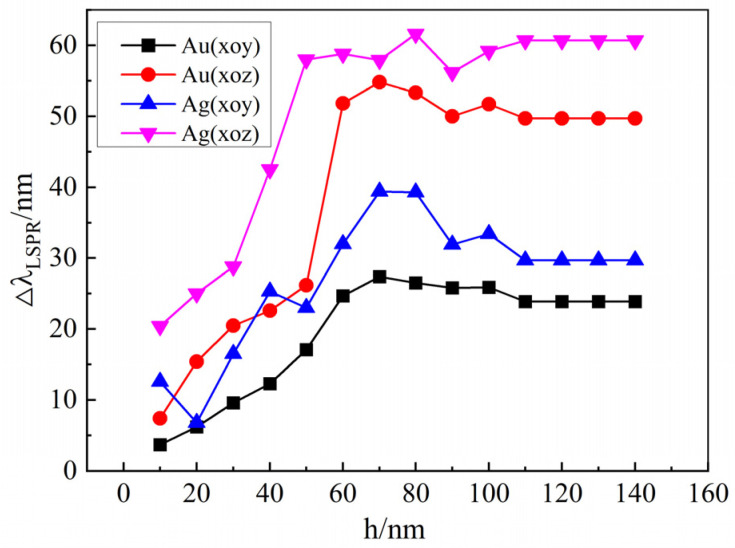
The tunability of the LSPR spectra for gold and silver array structures when the liquid crystal optical axis rotates within the xoy and xoz planes, respectively, for different liquid crystal layer thicknesses.

Figure 6 illustrates the tunability of the LSPR spectra for gold and silver array structures as the liquid crystal optical axis rotates in the xoy and xoz planes, respectively, at varying liquid crystal layer thicknesses. The array parameters are fixed at a = 50 nm, b = 80 nm, Px = Py = 40 nm, and T = 300 nm. It can be observed that the spectral tunability increases with liquid crystal layer thickness. However, beyond a thickness of 100 nm, the tunability saturates. Furthermore, comparison reveals that for a given noble metal nano-array under identical computational parameters, the LSPR tunability is higher during rotation within the xoz plane than within the xoy plane. Additionally, the silver nano-array exhibits greater tunability than the gold nano-array when the optical axis rotates within the same plane.

**Figure 7:**
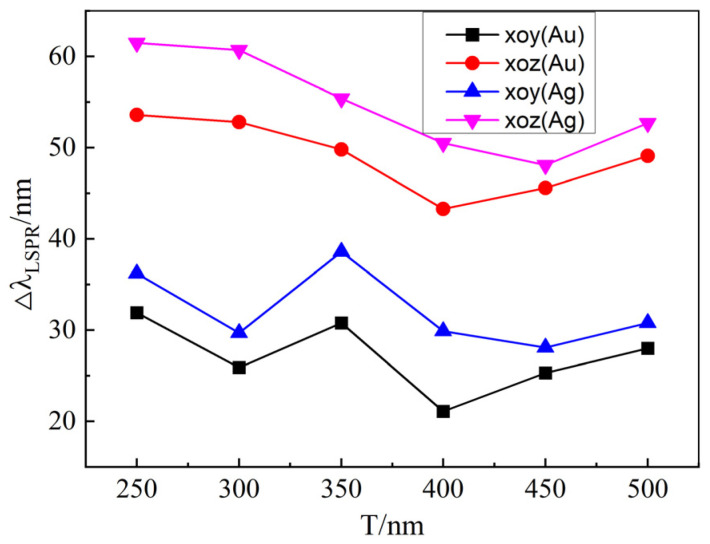
The tunability of the LSPR spectra for gold and silver array structures at different structural periods when the liquid crystal optical axis rotates within the xoy and xoz planes, respectively. The parameters for the structures are set as a = 50 nm, b = 80 nm, Px = Py = 40 nm, and h = 110 nm.

Figure 7 presents the tunability of the LSPR spectra for gold and silver nano-array structures at different structural periods when the liquid crystal optical axis rotates within the xoy and xoz planes, respectively. Clearly, with varying periods, the spectral tunability within the xoz plane remains higher than that within the xoy plane for the same array. However, the tunability variation with the period is unstable, which is attributed to changes in the local field distribution around the nano-array structures as the periods change. The interactions between metallic nanoparticles primarily comprise near-field and far-field interactions. Within a unit structure composed of four ellipsoidal metallic nanoparticles, near-field interactions dominate. Between distinct structures, far-field interactions dominate. When the periodic parameters are altered, the dominance of interactions between the metallic nanostructures shifts, leading to non-monotonic variations in the tunability of the LSPR spectra.

**Figure 8:**
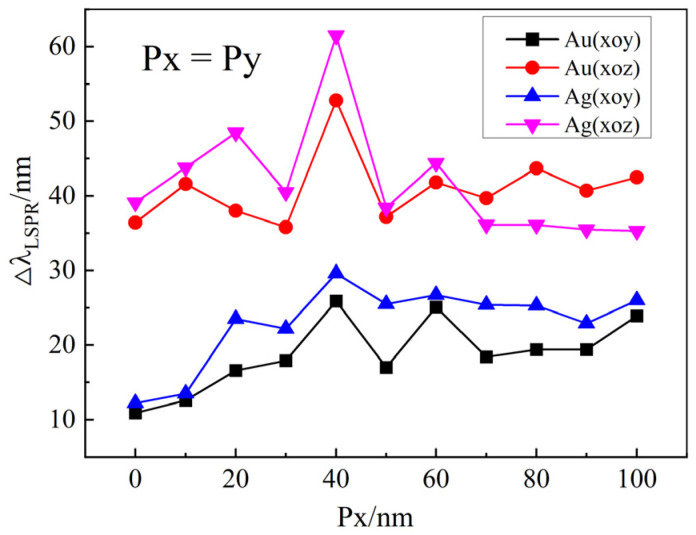
The tunability of the LSPR spectra for gold and silver array structures at different structure dimensions Px and Py when the liquid crystal optical axis rotates within the xoy and xoz planes, respectively. The parameters for the structures are set as a = 50 nm, b = 80 nm, T = 250 nm, and h = 110 nm.

Figure 8 illustrates the tunability of the LSPR spectra achieved by varying the structural dimensions Px and Py while keeping other parameters constant. Evidently, for both gold and silver nano-array structures, the LSPR spectral tunability within the xoz plane is consistently higher than that within the xoy plane. Moreover, the highest tunability occurs at Px = 40 nm. This optimal dimension enhances the interaction between incident light and the metal nanostructures, facilitating more efficient LSPR excitation.

## 4. Conclusions

In summary, this study investigates the extinction spectra of a novel noble metal nano-array structure within a liquid crystal environment. First, by varying the structural dimensions of the array, we achieved tunability of the localized surface plasmon resonance (LSPR) wavelength position. Second, through analysis of LSPR wavelength shifts during rotation of the liquid crystal optical axis within different planes (xoy and xoz), we established a direct correlation between LSPR spectral sensitivity and LSPR spectral tunability. This finding indicates that controlling the spectral tunability enables the modulation of spectral sensitivity. Finally, we compared the LSPR wavelengths and spectral sensitivities for arrays composed of gold versus silver. This study provides guidance for the future fabrication of photonic devices based on liquid crystal environments.

## Data Availability

The original contributions presented in this study are included in the article. Further inquiries can be directed to the corresponding authors.
